# Case report: Clinical and immunohistochemical manifestations of suspected Sjogren's disease in a dog

**DOI:** 10.3389/fvets.2024.1479363

**Published:** 2024-11-27

**Authors:** Brett D. Story, Sara M. Thomasy, Max W. Randolph, Anna Vincek, Bianca Martins, Erinn P. Mills, Jonathan D. Dear, Eric G. Johnson, Richard C. Jordan, Stephanie L. Goldschmidt, Natalia Vapniarsky

**Affiliations:** ^1^William R. Pritchard Veterinary Medical Teaching Hospital, School of Veterinary Medicine, University of California, Davis, Davis, CA, United States; ^2^Department of Surgical and Radiological Sciences, School of Veterinary Medicine, University of California, Davis, Davis, CA, United States; ^3^Department of Ophthalmology & Vision Science, University of California, Davis, Sacramento, CA, United States; ^4^Department of Medicine & Epidemiology, School of Veterinary Medicine, University of California, Davis, Davis, CA, United States; ^5^Departments of Orofacial Sciences, Radiation Oncology, and Pathology, and the Helen Diller Comprehensive Cancer Center, University of California, San Francisco, San Francisco, CA, United States; ^6^Department of Pathology, Microbiology and Immunology, School of Veterinary Medicine, University of California, Davis, Davis, CA, United States

**Keywords:** xerostomia, sicca, canine, Sjogren's disease, dry eye, keratoconjunctivitis sicca

## Abstract

Sjogren's disease, well-described in people, is rarely identified in veterinary species. In people, Sjogren's disease is one of the most common systemic autoimmune disorders with an incidence of 0.5% in the female population. The hallmark histopathologic finding of primary Sjogren's disease is lymphomononuclear cell infiltrates aggregating as periductal infiltrate in salivary glands. Sjogren's-like disease has been reported in a domestic shorthair cat and golden retriever dog. However, both lacked positive antinuclear antibody (ANA) titers and the dog showed no clinical evidence of dry eye disease. The following case report describes the clinical and immunohistochemical findings suggestive of Sjogren's disease in a 3-year-old spayed female German shepherd cross that was presented for medically refractory absolute dry eye, xerostomia confirmed with oral atropine response tests, and bilateral mandibular salivary gland enlargement. Routine topical lacrostimulants, anti-inflammatories, heterologous serum, ocular lubrication, and oral pilocarpine failed to improve clinical signs or tear production. The ANA titer at 1:160 was interpreted as positive, while the complete blood count and serum biochemistry panels were unremarkable. Head and neck ultrasound revealed bilateral moderately enlarged mandibular salivary glands with a hypoechoic, mottled echotexture consistent with sialoadenitis and regional lymphadenomegaly; thoracic radiography and abdominal ultrasonography were normal. *In vivo* confocal microscopy and spectral-domain optical coherence tomography of the cornea confirmed lipid keratopathy presumably secondary to corneal desiccation and steroid administration. Salivary gland histopathological and immunohistochemical analyses supported an immune-mediated etiology. Approximately 60% of the salivary section contained inflammatory cells replacing the glandular structures with a focus score of 12. Immunohistochemical markers CD3, CD204, CD79a, and CD20 were evaluated. The inflammatory infiltrate was a mixture of T-cells and macrophages with rare individual immunoreactive B-cells. CD3 and CD4+ T-cells were confirmed using immunohistochemistry and quantitative PCR, respectively. Clinical signs including ocular discharge and mandibular salivary gland enlargement markedly improved following oral immunomodulatory therapy with prednisone (1 mg/kg/d, tapered over 2 months) and long-term leflunomide (2 mg/kg/d). Ocular discomfort improved dramatically decreasing the need for topical lubricants; however, tear production failed to improve likely due to extensive lacrimal gland atrophy. The aim of this report is to increase awareness of Sjogren's disease in dogs and interpret the pathology involved.

## 1 Introduction

The prevalence of Sjogren's disease (SD) in veterinary medicine is disputed. In people, Sjogren's disease is a common systemic autoimmune disorder with an incidence of 0.5% in the female population (1). The disorder has been classically termed as primary (pSS) or secondary. When Sjogren's disease is accompanied by systemic lupus erythematosus, systemic sclerosis, or rheumatoid arthritis, it has been termed secondary, whereas pSS occurs without secondary tissue disorders. However, a push to unify the definitions of SD has recently been proposed (2). A diagnosis of SD is made utilizing multiple criteria established by an America-European consensus group, however; further validation and criteria for diagnosis are in development, and a clear consensus about the diagnostic criteria for pSS is debated (3, 4). Physical examination findings of keratoconjunctivitis sicca (KCS), xerostomia, and the presence of ANA antibodies generally support a SD diagnosis in humans. The histopathologic finding of lymphomononuclear cell infiltrates aggregating as periductal infiltrate in salivary glands of affected patients with primary Sjogren's syndrome (pSS) is a hallmark finding (5). The disorder can affect multiple secretory glands, resulting in fevers, pulmonary disease, salivary gland enlargement, as well as gastrointestinal, dermatologic, kidney, dental, rheumatologic, neurologic, and gynecologic disorders (6).

Previously, KCS and xerostomia were reported in two colony dogs (5 and 7-year-old female miniature poodles) with histopathology and ANA titers suggestive of SD (7). Sjogren's-like disease has also been reported in a domestic shorthair cat and golden retriever dog (8, 9). However, both clinical reports lacked positive ANA titers, and the dog showed no evidence of dry eye disease. The goal of this article is to further expand the knowledge of SD in veterinary medicine. Herein, we describe clinical and immunohistochemical findings in a 3-year-old spayed female German shepherd cross to help elucidate this potentially underreported disease in veterinary medicine.

## 2 Case report

A 3-year-old spayed female German shepherd cross weighing 33.4 kg (73.6 lb) was referred to the UC Davis William R. Pritchard Veterinary Medical Teaching Hospital (UCD-VMTH) for evaluation of KCS. The dog had a history of atopic dermatitis (3-month duration), intermittent antibiotic-responsive fevers since adoption (last fever 2 years prior to presentation), and bilateral ear infections. No recent history of viral infections, including COVID-19, were reported by the owners. Three months prior to evaluation at the UCD-VMTH, the dog presented to the primary veterinarian for conjunctival hyperemia in both eyes (OU); Schirmer tear test 1 (STT1) recordings were 0 mm/min OU. Treatment was initiated with a topical lubricant (I-Drop Vet Plus, I-Med Animal Health, Saint-Laurent, QC) OU q8hr, ofloxacin (0.3% solution; Bausch and Lomb, Laval, CA) in the left eye (OS) q8hr due to suspected superficial ulcerative keratitis, and Optimmune (cyclosporine 0.2% ointment; Merck, NJ, USA) OU q8hr. A referral examination by a veterinary dermatologist confirmed a diagnosis of atopic dermatitis, and subcutaneous Cytopoint injections (Zoetis Inc, NJ, USA) were recommended every 4–6 weeks with subcutaneous allergen injections every other week utilizing intradermal skin testing results. Two months prior to evaluation at UCD-VMTH, the dog was referred to a board-certified veterinary ophthalmologist due to severe blepharospasm and mucoid discharge OU; STT1 results were unchanged (0 mm/min OU). Tacrolimus (0.03% solution with aqueous; Stokes Pharmacy, NJ, USA) OU q12hr, Neomycin-polymyxin-dexamethasone ointment (Bausch and Lomb, Laval, CA) OU q24hr and topical lubrication with Optixcare as frequent as possible OU (CLC Medica, Ontario, CA) were recommended. At this visit, oral atropine (1% solution; Apotex, Toronto, CA) was administered to determine candidacy for parotid duct transpositions (PDT) OU. Oral application of topical atropine typically leads to profuse immediate salivation due to its bitter taste. No salivary production was appreciated, and a guarded prognosis for PDT was given. Subsequent referral to the UCD-VMTH was recommended by the primary veterinary ophthalmologist.

**Figure 1:**
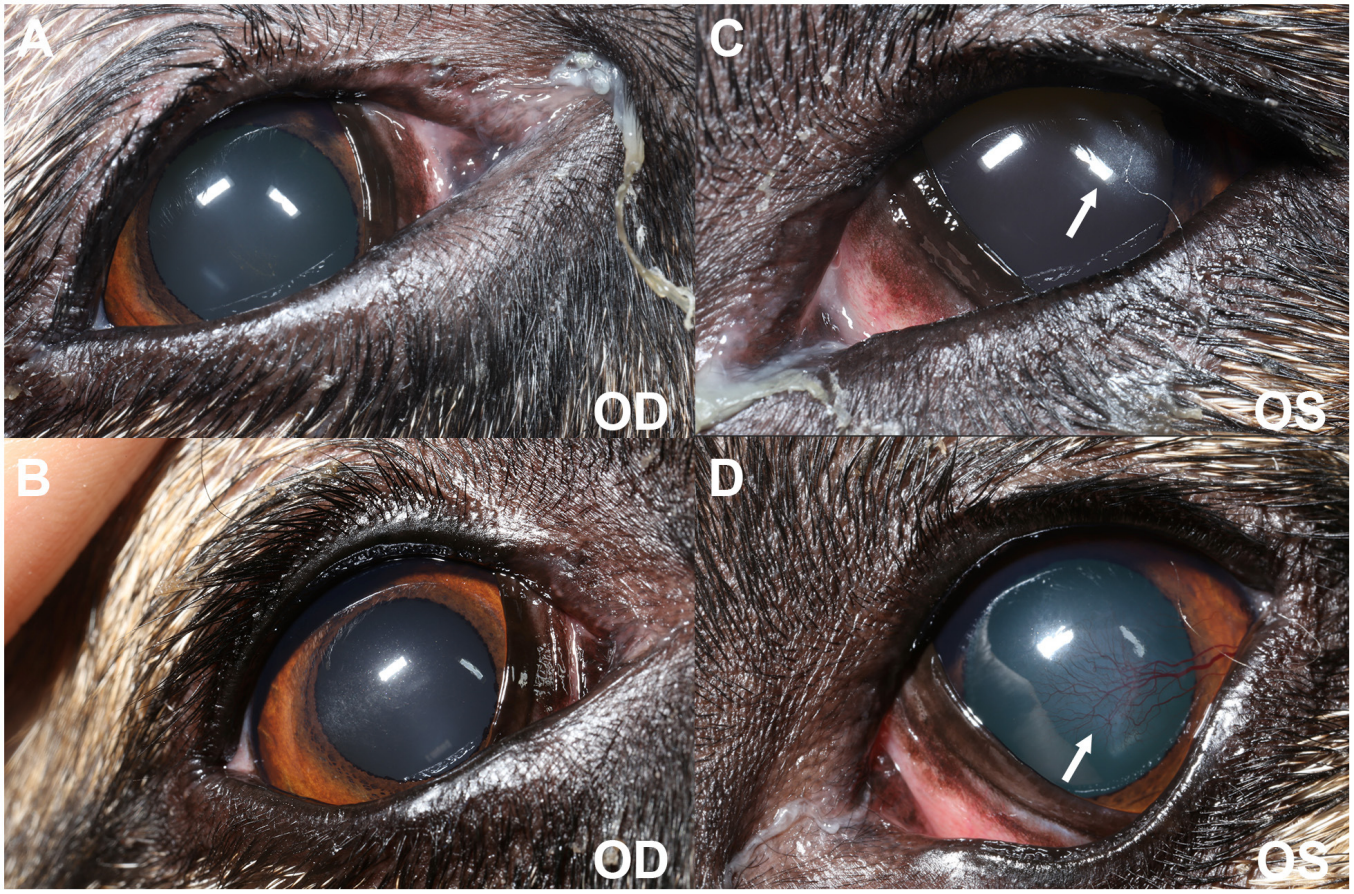
Clinical photography at initial evaluation **(A, C)** and 6-months post oral anti-inflammatory therapy **(B, D)**. **(A, C)** Blepharospasm is evident from the decreased interpalpebral fissure opening OU (OS > OD). There is mild to moderate mucoid discharge present at the medial canthus OU. Diffuse conjunctival hyperemia of the anterior surface of the third eyelids is observed OU. A focal ~3 mm diameter region of subepithelial ovoid superficial corneal haze is seen OS (white arrow) in the axial to temporal paraxial cornea. **(B, D)** Blepharospasm is absent OU. There is mild mucoid discharge at the medial canthus OU. Conjunctival hyperemia has improved OU. Mild axial (OD) and temporal paraxial (OS) corneal fibrosis remains with improvement in density OS and new superficial corneal vascularization (white arrow).

On presentation to the UCD-VMTH, the dog was bright, alert, and responsive. The general physical examination, including mandibular salivary gland and submandibular lymph node palpation was unremarkable. Adnexal examination revealed mild to moderate blepharospasm OU. A thick, mostly crusted, mucoid discharge was appreciated OU. Neurophthalmic examination, including visual function in photopic conditions, was normal. Examination of the conjunctiva and anterior segment revealed mild diffuse conjunctival hyperemia OU, lackluster precorneal tear films OU, mild axial subepithelial to anterior stromal corneal haze OS (~3 mm diameter), and pulverulent nuclear cataracts OU (Figure 1). STT 1 (Merck, NJ, USA) was 0 mm/min OU, intraocular pressures with rebound tonometry (TonoVet; Jorgensen Laboratories, CO, USA) were 14 mmHg in the right eye (OD) and 13 mmHg in the left eye (OS). Both nares were moist, indicating that xeromycteria was not present. Fluorescein staining (BioGlo; HUB Pharmaceuticals, MI, USA) of the cornea showed mild, diffuse multifocal punctate stain OU consistent with corneal desiccation with no epithelial loss. The dog was continued on topical tacrolimus 0.03% aqueous solution and neomycin-polymyxin-dexamethasone ointment as previously prescribed. Pilocarpine (0.1% solution; Stokes Pharmacy, NJ, USA) OU q8hr and 1.2% hyaluronate (An-HyPro; An-Vision, Henningsdorf, GER) OU q4-6hr were added.

One month later, the dog presented for a recheck examination. The owners continued to report squinting (OS worse than OD) and increased redness OU. The owner reported that 2 weeks prior, the dog had developed two large masses around her neck which were biopsied by the primary veterinarian with inconclusive results. Doxycycline (12 mg/kg) PO q24hr was started by the referring primary veterinarian, but no improvement was observed. Topical pilocarpine was discontinued due to ocular irritation. On clinical examination, STT1 remained static at 0 mm/min OU. Ophthalmic findings were similar to the initial examination, except new diffuse axial subepithelial to anterior white crystalline stromal opacifications were identified as consistent with lipid deposits from steroid keratopathy OU. Oral atropine administration resulted in no salivation. On physical examination, palpation of the neck revealed bilateral mandibular salivary gland enlargement (left greater in size compared to right). The remainder of the physical examination, including peripheral lymph node palpation, was unremarkable. Previous bloodwork provided by the primary veterinarian 2 months prior showed a leukocytosis characterized by neutrophilia (19 K/uL; RR 2.94–12.67), mild hypokalemia (3.8 mmol/L; RR 4.0–5.4), with normal urinalysis, and normal thyroid function (2.0 μg/dL; RR 1.0–4.0). Given the refractory state of the patient's ocular disease, tacrolimus was increased in concentration (1% aqueous solution) and frequency (q8hr), heterologous canine serum was administered OU q8hr to aid in ocular discomfort, (10) an artificial ointment (Soothe, Bausch and Lomb, Laval, CA) was recommended in the evening, and pilocarpine (2% ophthalmic solution; Sandoz, Basel, CH) drops were administered in food at a frequency under which parasympathetic clinical signs (e.g., hypersalivation, diarrhea, and vomiting) were not appreciated. Considering all findings combined, parotid duct transposition was discussed with a poor prognosis for success.

**Figure 2:**
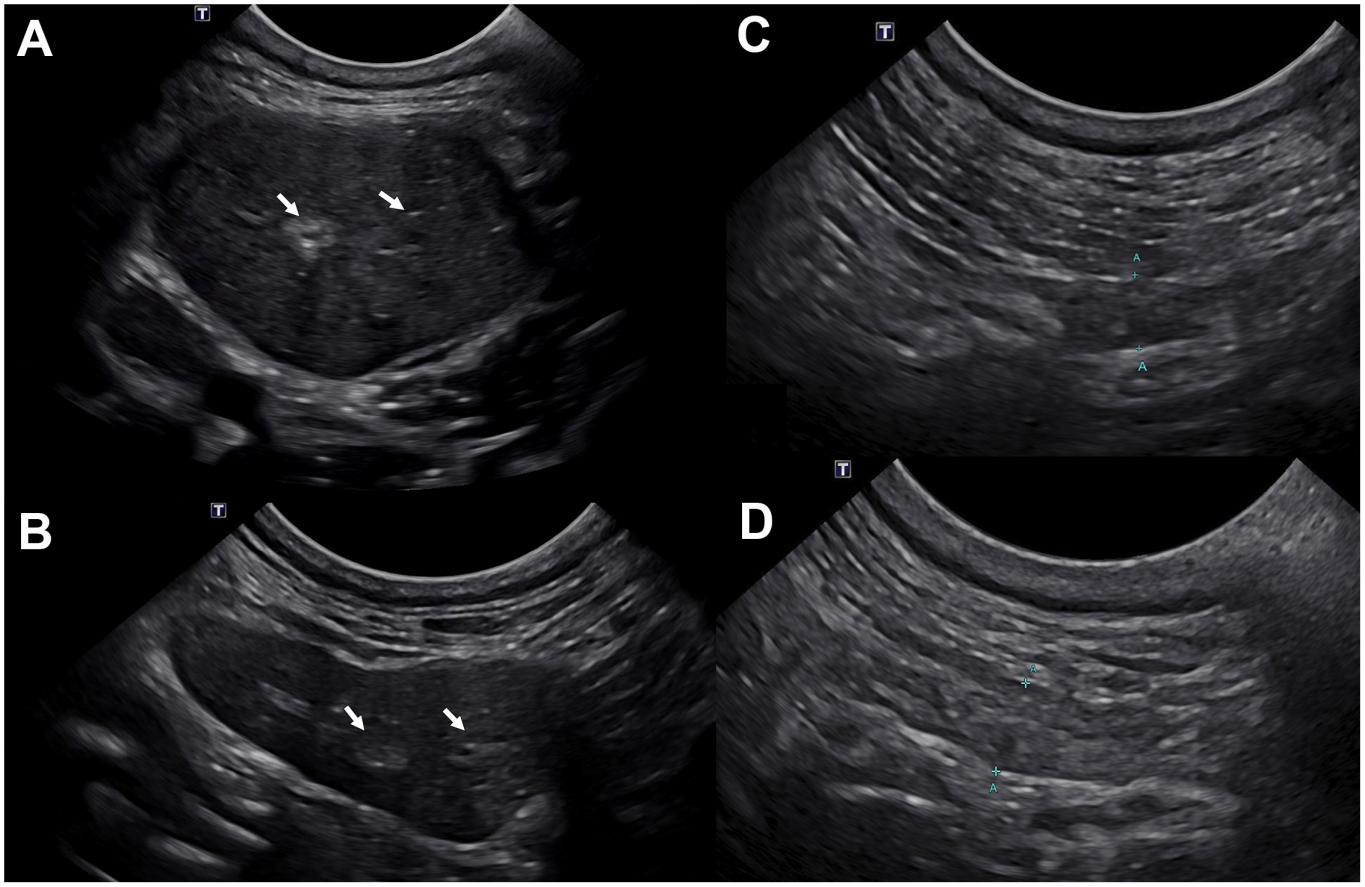
Ultrasonographic images of the left mandibular **(A, B)** and left parotid **(C, D)** salivary glands demonstrate reduced size and increased echogenicity following marked improvement prior to **(A, C)** and following **(B, D)** oral anti-inflammatory therapy. **(A)** At initial examination, both mandibular salivary glands were moderately enlarged (left shown only) and hypoechoic with a subtle hypoechoic mottle; mild hyperechogenicity of the fat surrounding the salivary glands was observed. The parenchyma was subtly heterogenous (white arrows). The left mandibular salivary gland measured ~2.5 cm in height. **(B)** The size of both mandibular salivary glands (left shown only) was within normal limits 3 months after initiating oral anti-inflammatory therapy. The parenchyma remains subtly heterogeneous with few hyperechoic regions and speckles throughout (white arrows). The tissue surrounding the salivary glands is now quiescent consistent with improved yet mildly persistent bilateral mandibular parenchymal change without current evidence of active inflammation. The left mandibular salivary gland measured 1.2 cm in height. **(C)** Mild altered echogenicity of the left parotid gland with few hypoechoic parenchymal regions was observed at initial examination. The left parotid gland measured ~0.5 cm in height. **(D)** Mildly improved left parotid gland echogenicity 3 months after initiation of oral anti-inflammatory therapy. The left parotid gland measured ~0.45 cm in height. The changes in the parotid glands were less pronounced compared to the mandibular salivary glands.

Given the absolute KCS, xerostomia, and new development of bilateral salivary gland enlargement, SD was suspected and detailed diagnostic testing at the UCD-VMTH was performed. Thoracic radiography and abdominal ultrasonography were unremarkable. Head and neck ultrasound of the parotid and mandibular salivary glands was performed using a Canon Aplio i800 i-series ultrasound system (Canon Medical Systems USA Inc., 2441 Michelle Drive Tustin, CA 92780) with an 11MC4 multi-frequency microconvex transducer (center frequency = 7 MHz). Ultrasonography revealed sonographic changes to the mandibular and parotid salivary glands and regional lymphadenopathy (Figure 2). The mandibular salivary glands were bilaterally moderately enlarged (2.5 cm in height) and hypoechoic with subtle hypoechoic mottling. There were few small hyperechoic foci within the salivary glands bilaterally, without distinct distal acoustic shadowing. The fat surrounding the mandibular salivary glands was hyperechoic. The parotid salivary glands were bilaterally mildly hypoechoic with subtle hypoechoic mottling. They measured approximately 0.5 cm in height bilaterally. The medial retropharyngeal, mandibular, and parotid lymph nodes were bilaterally mildly rounded with ill-defined intraparenchymal hypoechoic regions. They measured normal to minimally enlarged; the maximal height was measured at the right lateral mandibular lymph node (~0.55 cm in height). The thyroid glands were normal. Differential diagnosis for these changes included non-infectious inflammatory (SD, immune mediated), round cell neoplasia (lymphoma), and infectious inflammatory etiologies. Imaging with computed tomography to evaluate the zygomatic salivary glands and lachrymal glands was declined by the owners. *In vivo* confocal microscopy (IVCM; ConfoScan 4, Nidek, Japan) and spectral-domain optical coherence tomography (SD-OCT; Spectralis^®^ HRA + OCT, Heidelberg, Germany) demonstrated bilateral axial corneal subepithelial to stromal corneal hyperreflective deposits consistent with lipid secondary to topical steroids. Antinuclear antibody (ANA) titer was performed and interpreted as positive at 1:160.

**Figure 3:**
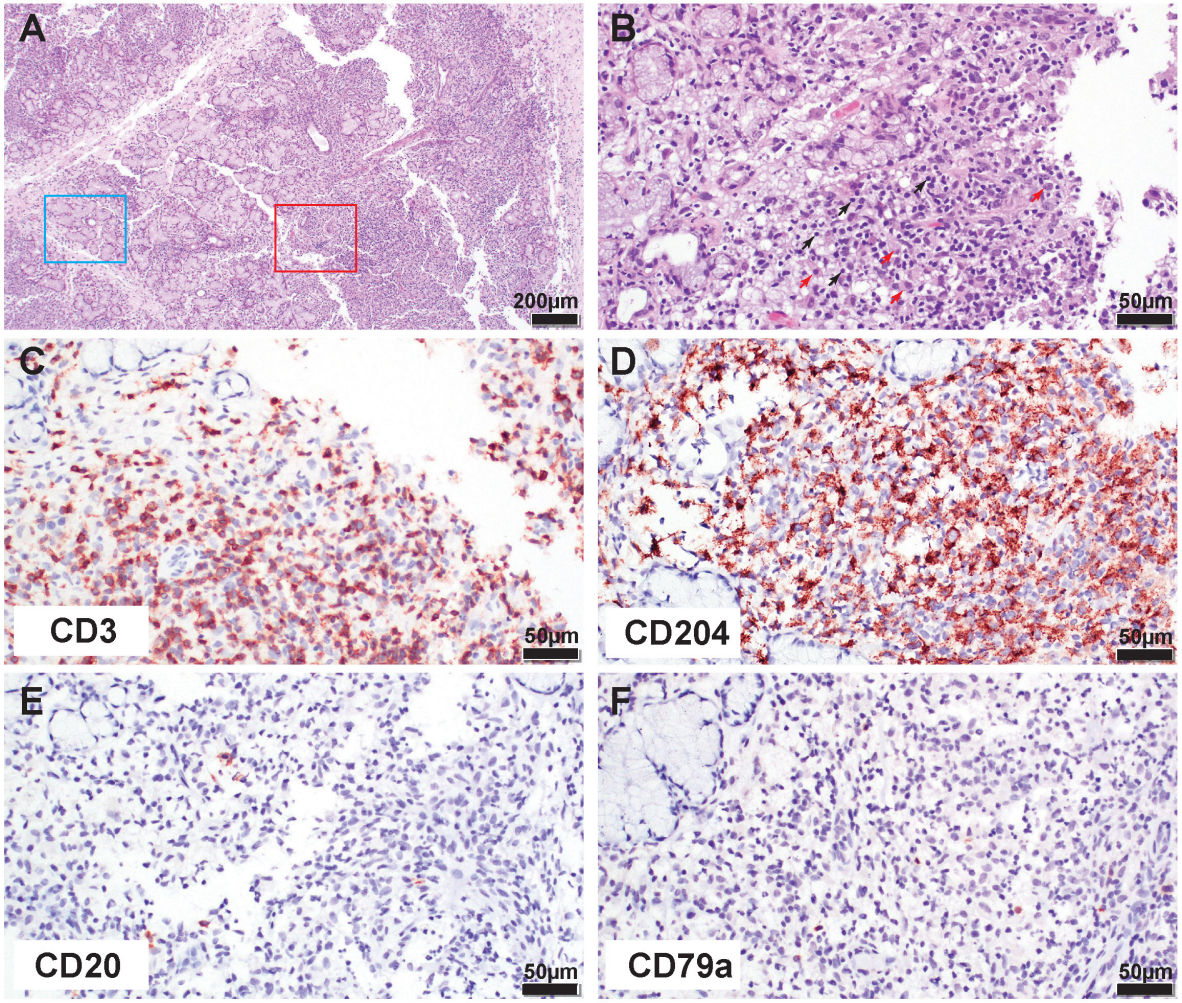
Histological and immunohistochemical (IHC) appearance of the mandibular salivary gland demonstrates lymphohistiocytic inflammation consistent with SD. **(A)** Low magnification of the inflamed salivary gland. Note, normal tubuloacinar architecture still remaining (blue rectangle) while approximately 60% of the section is heavily infiltrated by inflammatory cells replacing and effacing the glandular structures (red rectangle). **(B)** At higher magnification note the predominance of small lymphocytes (black arrows) and histiocytes (red arrows) in the inflammatory infiltrate. **(C)** IHC for CD3 antigen. Note that approximately 50% of the inflammatory infiltrate is immunoreactive for CD3 (T cells). **(D)** IHC for CD204 antigen. Note that approximately 50% of the inflammatory infiltrate is immunoreactive for CD204 (macrophages). **(E)** IHC for CD20 antigen. Note rare individual immunoreactive B cells scattered throughout the section. **(F)** IHC for CD79a antigen. Note rare individual immunoreactive B cells scattered throughout the section.

The previous mandibular salivary gland biopsies were acquired from the primary veterinarian for further review by board-certified veterinary and human medicine oral pathologists at UC Davis and UC San Francisco, respectively. Both histopathology and immunohistochemistry (IHC) were performed on the salivary gland biopsy; 41 mm^2^ of salivary tissue was available for analysis. Approximately 60% of the salivary gland section was heavily infiltrated by inflammatory cells, predominantly lymphocytes and histiocytes, which replaced and effaced the glandular structures (Figure 3). There were confluent foci of lymphocytes, and a focus score of 12 was assigned. In humans, histological diagnosis of pSS relies heavily on the observation of at least one focus, which is an aggregate of ≥50 mononuclear cells surrounding a vessel or duct within 4 mm^2^ of glandular tissue (focus score ≥1) (4). Furthermore, there was no evidence of neoplastic (lymphoma) or infectious agents. Paraffin embedded salivary gland tissue was stained with CD3 (CD3-12, rat polyclonal, Moore Lab, 1:10 dilution), CD204 (SRA-E5, mouse polyclonal, Trans Genic Inc, Kobe, JPN, 1:200 dilution), CD20 (SP.32, rabbit polyclonal, Abcam, Cambridge, UK, 1:100 dilution), and CD79a (HM57, mouse polyclonal, Bio-Rad, CA, USA, 1:100 dilution). Antigen retrieval was performed with a citrate buffer or EDTA9 (Dako S2368) steam for 30 min. Immunohistochemical analyses for antigens CD3 and CD204 showed that the cellular infiltrate consisted of T cells and macrophages at a 50:50 ratio (Figure 3). Additional IHC testing for antigens CD20 and CD79a revealed rare individual B cells that were scattered randomly throughout the salivary gland section. Given the inflammatory infiltrate in human cases of pSS is dominated by CD4+ T cells, (11) quantitative real-time PCR (q-RTPCR, primer Cf02627844_m1, ThermoFisher, CA, USA) was performed. The q-RTPCR results (Cq values) were compared between the dog's salivary gland biopsy (27.04) and normal canine mandibular lymph node tissue (27.39). Provided the normal salivary gland is expected to contain minimal to no resident lymphocytes; this outcome indicates extremely high prevalence of CD4 lymphocytes in the gland, similar to that of lymphoid tissue (Supplementary Data 1).

**Figure 4:**
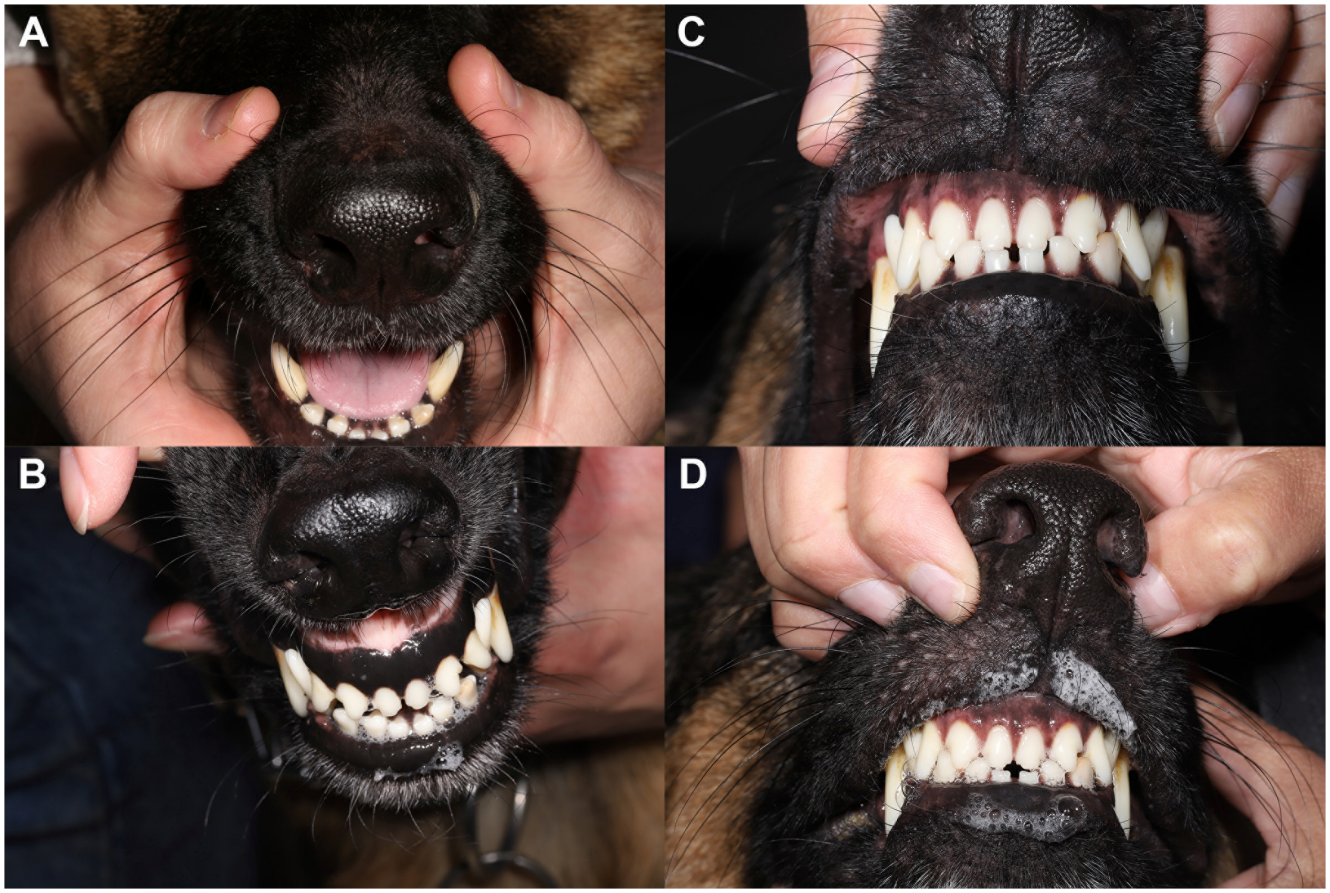
Atropine oral response test demonstrates marked improvement following initiation of oral anti-inflammatory medication. **(A)** Persistence of xerostomia despite application of topical atropine 1% solution per os prior to initiating oral anti-inflammatory therapy. **(B)** A normal atropine response with mild to moderate salivation returned following 1 month of oral anti-inflammatory therapy. Pre **(C)** and post **(D)** atropine oral response test 6-months following oral anti-inflammatory medication. Xeromycteria was not appreciated at any examination as indicated by moist nares in all photos.

Oral pilocarpine was discontinued due to lack of clinical response, and oral prednisone (1.3 mg/kg/day) was initiated. One month following oral anti-inflammatory steroid administration, the dog had markedly improved with resolution of the salivary gland swelling. The dog's blepharospasm had subsided with minimal residual conjunctival hyperemia and discharge, as appreciated by the owners. Given the positive response to oral prednisone, the dose was tapered, and oral leflunomide (2 mg/kg/day, Stokes Pharmacy, NJ, USA) was started for long-term therapy. At the next follow-up 1 month later (day 282 post-initial presentation), the dog's eyes were open and comfortable with mild ocular discharge OU. Teriflunomide, the active metabolite of leflunomide, serum concentrations were 25.1 μg/mL, and within a range (25–45 μg/mL) expected to control immune-mediated disease in dogs. Despite the marked improvement in ocular comfort, tear production as measured by STT1 remained 0 mm/min OU. However, a positive response to oral atropine was appreciated, indicating salivary production was regained (Figure 4). Repeat ultrasonography of the salivary glands revealed normal mandibular and parotid salivary gland size with mild parenchymal remodeling; no evidence of active inflammation was observed (Figure 2). Specifically, the parenchyma of these glands remained subtly heterogeneous, with a few hyperechoic regions and speckles throughout. A similar but decreased regional lymph node change was still present. In aggregate, these findings were consistent with a positive response to treatment. Six months post-initiation of oral immunosuppressives, the dog's eyes remained open and comfortable with minimal ocular discharge OU (Figure 1).

## 3 Discussion

The present study documents the clinical, ultrasonographic, histological, and immunohistochemical findings of suspected SD in a 3-year-old spayed female German shepherd cross. The diagnosis of dry eye was confirmed with STT1 results and ocular clinical signs. The evidence of xerostomia was obtained by lack of salivary production post oral atropine response testing. Furthermore, the immune-mediated etiology of the salivary gland inflammation was determined via histology, immunohistochemistry, molecular tests, and a positive ANA titer. Salivary glandular ultrasonography further characterized the disease process and therapeutic response. Finally, the SD diagnosis was reaffirmed by the patient's favorable response to immunosuppressive therapy, resulting in a marked improvement of both dry eye and xerostomia symptoms. To the author's knowledge, this is the first canine case presented in the literature with essential features of SD, thus expanding the knowledge on this rare veterinary disease.

In general, a diagnosis of SD in human patients is made based on the findings of KCS, xerostomia, a positive ANA titer, histopathology, and the presence of autoantibodies (Table 1) (12). The diagnosis can be complicated in patients with mild sicca symptoms or less characteristic antibody profiles. An international consensus group (3) has suggested criteria for the diagnosis of pSS in humans that require four of six criteria (3), including a positive minor-salivary-gland biopsy sample (focus score > 1) or autoantibody reactivity, at least one ocular and oral symptom, evidence of dry eye disease, and diagnostic evidence of salivary gland involvement. Recently, three novel autoantibodies were identified to assist in the diagnosis of SD in humans- anti-salivary protein 1, anti-parotid secretory protein, and anti-carbonic anhydrase VI (13). While these specific autoantibodies are not available in veterinary medicine, ANA titer, if interpreted cautiously, can aid in the conformation of SD-like and SD diagnoses. However, positive ANA titers in dogs can also occur in a variety of diseases such as inflammatory disorders (Doberman hepatitis, atopic dermatitis, systemic lupus erythematosus), infectious diseases (Leishmaniasis, Ehrlichiosis), those related to medication administration (tetracyclines, penicillin, sulfonamides), or neoplasia. The patient reported here exhibited no major or minor clinical signs associated with systemic lupus erythematosus or other entities listed, suggesting that positive ANA titer was likely due to SD, however; a positive titer secondary to atopic dermatitis cannot be ruled out. Quantitative dry eye and xerostomia have both been described in two canine patients by Quimby et al. (7), in which tear production, as measured with STT1, ranged from 1–10 mm/min. Both dogs had clinical evidence of an extremely dry mouth with necrotizing gingivitis. Also, SD-like was recently described by Nabeta and colleagues in a neutered male Golden Retriever of unknown age with a chief complaint of xerostomia, but ocular clinical signs were lacking (8). In humans, approximately 20% of Sjogren's patients do not have sicca symptoms (14), which suggests that SD presentation may have a spectrum of symptoms of various extents and severity. Similarly, in reported canine cases, palpably enlarged mandibular salivary glands were present in one study (8) but absent in the other (7). In conclusion, similar to humans, the diagnosis of SD or SD-like in canine patients must rely on constellations of symptoms and should be supported by multiple diagnostic tests.

**Table 1:** Human international consensus (3, 12) and veterinary suggested consensus for diagnosis of Sjogren's disease.

**Human international consensus for Sjogren's disease**	**Suggested veterinary diagnostic criteria for Sjogren's disease**
**Ocular symptoms (at least one present)**	**Ocular symptoms (at least two present)**
Dry eyes for >3 months	Blepharospasm
Sensation of sand or gravel in eyes (recurrent)	Mucoid to mucopurulent ocular discharge
Use of tear substitutes > 3x per day	Conjunctival hyperemia
**Oral symptoms (at least one present)**	Lackluster corneal surface
Daily feeling of dry mouth >3 months	Superficial corneal vascularization
Recent or persistent swollen salivary glands as adult	**Oral symptoms (at least one present)**
Frequently drinking liquid to assist swallowing dry foods	Abnormal oral atropine response test
**Evidence of dry eye (at least one abnormal result)**	Dry, sticky gums
Schirmer Tear Test 1	Thick pasty or absent saliva
Rose Bengal staining	Halitosis
**Histopathology**	Swollen salivary glands
Focal lymphocytic sialoadenitis in a minor salivary gland with a focus score ≥1 • Defined as the number of lymphocytic foci per 4 mm^2^ of glandular tissue	**Evidence of dry eye (at least one abnormal result)**
	Schirmer Tear Test 1
**Salivary gland involvement (at least one abnormal result)**	Rose Bengal staining
Salivary scintigraphy	Lissamine green staining
Parotid sialography	**Histopathology**
Unstimulated salivary flow	Focal lymphocytic sialoadenitis in a minor salivary gland with a focus score ≥1 • Defined as the number of lymphocytic foci per 4 mm^2^ of glandular tissue
**Laboratory abnormalities (at least one present)**	
Antibodies to Ro(SSA) or La(SSB) antigens, or both	Immunohistochemistry • T-cell inflammatory infiltrate
ANA Titers	
IgM rheumatoid factor (anti-IgG Fc)	**Salivary gland involvement (at least one abnormal result)**
	Salivary gland ultrasonography
	Computed tomography
	**Laboratory abnormalities (+/-)**
	ANA Titers

Ultrasonographic findings in Nabeta and coauthors report on SD-like disease in a Golden Retriever (8), which mirrored the findings in the present study. Specifically, multifocal hypoechoic areas appreciated in the glandular parenchyma were similar features. A limitation of the current study was the lack of computed tomography (CT). The CT findings in the Nabeta et al. study included multiple and bilateral enlargement of the salivary glands, which included the mandibular, parotid, and zygomatic glands, with additional enlargement of the lacrimal and third eyelid glands (8). In the present case, parotid and mandibular salivary gland enlargement were manually palpated and observed with head and neck ultrasonography, but zygomatic, lacrimal, and third eyelid glandular changes were not evaluated. We agree with Nabeta et al. that both salivary gland head and neck ultrasonography and CT of the head and neck are key diagnostic tools in the diagnosis and response to therapy of Sjogren's cases.

Classically, in humans, the hallmark finding pathologically in SD is a chronic cellular infiltrate of mononuclear cells, with the majority (>75%) of lymphocytes infiltrating the salivary glands identified as CD4+ T cells (15). More recently, macrophages have been identified to play a critical role in the pathogenesis of pSS (16). In a murine model of SD, CD4+ T cells and macrophages are in close proximity, suggesting an interaction between these two cell types in diseased tissue (17). In human reports, the percentage of macrophage infiltration varies greatly, likely due to disease stage differences and/or in IHC markers and diagnostic approaches to identify these cells (18, 19). However, the incidence of macrophage infiltration positively correlates with lesion grade, focus biopsy score, and correlates with degree of salivary gland enlargement (20, 21). Staining for B cells is also important for suspected cases as evolution into B cell lymphoma is one of the major risk factors for survival in pSS and occurs in about 5% of human patients (22). In our case, the standard histopathology of the salivary gland biopsy demonstrated robust lymphohistiocytic inflammation of the gland, effacing and disrupting the normal architecture of the ducts and acini. The predominant cell types in the inflammatory infiltrate were T cells (CD3 immunoreactivity) and macrophages or histiocytes (CD204 immunopositivity), while the prevalence of B cells (CD20 and CD79a immunoreactive cells) was rather low (Figure 3). Recently, the roll of B-cells in the pathogenesis of human SD is being acknowledged, potentially offering therapeutic implications (23). The low number of B-cells in our case may be attributed to interindividual or interspecies variations in SD pathogenic mechanisms. q-RTPCR results further confirmed the presence of CD4+ T cells in the inflammatory infiltrate. Together, these observations indicate an inflammatory etiology and exclude a diagnosis of lymphoma. Our case findings mirror the reports by Quimby et al. and Nabeta et al., where lymphocyte infiltration was documented. To the best of our knowledge, IHC workup was not performed in any of the existing reports in dogs (7, 8). Additionally, in human medicine, histology is required for a focus score assignment. A focus score >1 is one of the most important tests in the diagnosis of human oral SD and is defined as at least 50 mononuclear inflammatory cells in a 4 mm^2^ glandular histology section (24). In our 41 mm^2^ section of glandular tissue, a focus score of 12 was assigned, which is consistent with SD. Provided that the demonstration of lymphocytes and histiocytes is essential for the conformation of the SD diagnosis, we posit that histology and IHC are essential components of the diagnostic workup of the SD and SD-like cases.

Treatment of human cases of SD often involves the use of glucocorticoids, particularly for glandular enlargement (25). In the present case, no ocular response was obtained with topical serum, topical immunosuppressants and lacrostimulants (cyclosporine and tacrolimus), or oral or topical pilocarpine. Ophthalmic lubricants also failed to improve ocular comfort until oral anti-inflammatory therapy was started. Glucocorticoids were initially chosen due to their rapid onset of action, and a dramatic improvement was observed. Due to the long-term side effects of oral prednisone therapy, leflunomide was added for maintenance therapy as this is generally safe and well tolerated in dogs long-term, with the added benefit of therapeutic drug monitoring (26). Despite a dramatic improvement in ocular comfort, STT1 remained at 0 mm/min OU. We presume this is secondary to marked lacrimal gland destruction and/or atrophy from the chronic disease course and the improvement in patient comfort is owed to the anti-inflammatory effects of the prescribed oral medications. Similarly, pharmacologic immunosuppression failed to alleviate xerophthalmia in a reported feline case (9). Prednisone induction and maintenance therapy with azathioprine led to improved clinical signs in Sjogren's-like disease in a Golden Retriever (8). Given the resolution of salivary gland enlargement and salivary production observed following lingual application of atropine with immunosuppression, a PDT with a fair prognosis could be considered for this patient if dry eye clinical signs worsen.

## 4 Conclusion

Sjogren's disease is rarely identified in veterinary species. Head and neck ultrasound and histopathology with focus scoring are valuable diagnostics for achieving a diagnosis. Immunohistochemistry further aids in the diagnosis and characterization of canine SD. Tear production may fail to improve with therapy, particularly with chronic disease. Oral anti-inflammatory glucocorticoids were pivotal in improving ocular comfort, glandular enlargement, and xerostomia; these were sustained by long-term immunosuppression with leflunomide. A diagnosis of SD should be considered in veterinary patients with bilateral mandibular salivary gland enlargement, xerostomia, and bilateral KCS with positive ANA titers. T cells and macrophages may be identified on IHC of glandular biopsies. Further histopathological studies in suspected canine patients may help elucidate the pathogenesis of SD in dogs and may assist with prevention, diagnosis, and treatment of this fascinating disorder.

## Data Availability

The datasets presented in this study can be found in online repositories. The names of the repository/repositories and accession number(s) can be found in the article/Supplementary material.
